# Molecular Diagnostics with Laboratory Finding in Patients with Myeloproliferative Neoplasms

**Published:** 2017-11

**Authors:** Zahra MAKANI, Sina MIRZAAHMADI, Marzieh MOTALLEBI, Abolfazl MOVAFAGH

**Affiliations:** 1. Dept. of Biology, Zanjan Branch, Islamic Azad University, Zanjan, Iran; 2. Dept. of Medical Genetics, School of Medicine, Cancer Research Center, Shahid Beheshti University of Medical Sciences, Tehran, Iran

## Dear Editor-in-Chief

According to WHO Classification System for Myeloproliferative Neoplasms (MPNs), *JAK2 V617F* mutation in exon 12 in hematopoietic lineages was established in MPNs disorders (1).

The discovery of the *JAK2V617F* mutation and recent advances in genetic basis of the Philadelphia (Ph) chromosome-negative (2) (MPNs) essential thrombocythemia (ET), Polycythemia Vera (PV), and myelofibrosis (MF) have made a significant contribution to understanding the pathogenesis of myeloproliferative neoplasms (3).

Among the cases of PV negative for the V617F mutation, several mutations in exon 12 are found in a significant number of cases (3,4). Ten various sequence differences have been studied, most of them is in the address gene between 536 and 544 codons (4). The novel mutation is reported as *N542-E543del* and *E543-D544del JAK2 617* mutation in exon 12 in the affected patients. However, some patients with MPNs disorder did not exhibit mutation *JAK2* in exon 12 (5).

Our aim of this study was to verify the relationship between N542-E543del mutation of JAK2 gene and MPNs in V617F-negative patients. The subjects were enrolled from some clinics in Babol, Mazandaran, northern Iran from March 2014 to June 2015. Forty-four healthy volunteers (23 males and 21 females, aged from 35 to 67), and 34 patients with MPNs (19 males and 15 females, aged from 40 to 72), were recruited and were consistent with Hardy Weinberg equilibrium. Genomic DNA was extracted from peripheral blood using the QIAamp Qiagen protocol.

The samples were collected from subjects after written informed consent was obtained in accordance with the Declaration of Helsinki (1). The study was approved by the Ethics Committee of the university.

The PCR-amplified products were detected in gel electrophoresis apparatus. The DNA molecular weight marker with instruction (Fermentas, Germany) was treated as a marker level size. The electrophoresis was done and images were selected in doc gel UVI documentation protocol (England). After Analysis *JAK2V617F* Mutation, the results were employed with *t*-test using SPSS Ver. 13 (Chicago, IL, USA).

In all cases, PCR-digestion analysis confirmed the allele-specific PCR results. Furthermore, PCR products for digestion analysis were purified by concert rapid PCR purification system (GibcoBRL, UK; Lark Technologies, Inc).

**Fig. 1:**
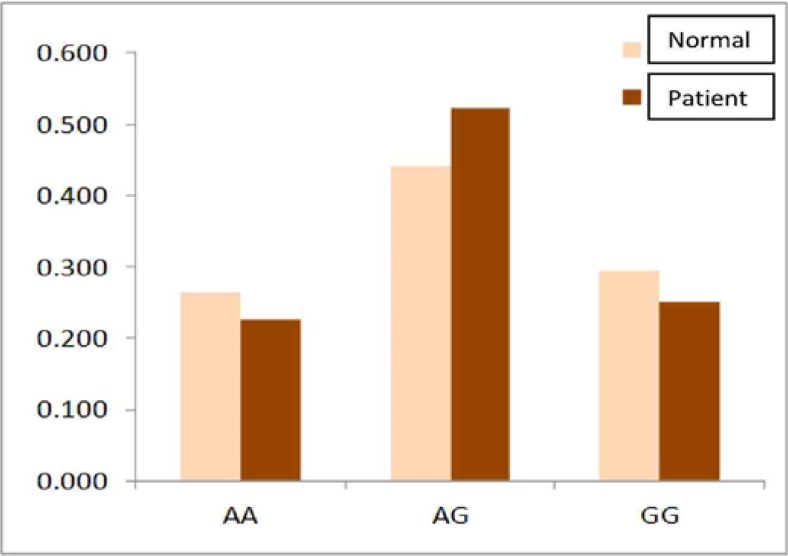
Genotype frequency of rs*7869668* between patients and normal controls

In this case-control study, there was no significant difference between the patients and controls groups by Pearson chi square analysis in geno-type distribution of the frequency of single nucleotide polymorphism rs7869668 of *JAK2* exon 12 (*P* >0.05). In addition, genotype analysis revealed that the patients were negative for the *JAK2-V617F* mutation (Fig. 1).

Mutation of V617F does not play a significant role in the pathogenesis of multiple myeloma (6). It is corresponding to our result that mutation of substitution of valine to phenylalanine at amino acid position 617 did not occure. Mutations in exons 12 and myeloproliferative neoplasms are reported (7).

Patients with a JAK2 exon 12 mutation with a hematologic disorder consistent with the diagnosis of PV is characterized by erythrocytosis, with a raised hematocrit and hemoglobin level (8). Unlike Williams et al. study, the mutations in exons 12 including *N542-E543del* was observed in no patient of the present investigation (9). Several somatic mutations of *JAK2* exon 12 can be found in MPDs mainly characterized by erythrocytosis. Moreover, a genetic predisposition to acquisition of different *JAK2* mutations is inherited in families with MPDs (5).

In this study, a correlation was applied in which the *JAK2-V617F* mutation may be useful for management of patients and classification with the MPDs. However, the clinical and genotyping of finding a mutation and non-significant correlation between patients and controls group in this study in such small fraction of the population is understood.
